# Tourniquets for the control of traumatic hemorrhage: a review of the literature

**DOI:** 10.1186/1749-7922-2-28

**Published:** 2007-10-24

**Authors:** Stephen L Richey

**Affiliations:** 1 CRT Saginaw Valley State University, University Village 453-5D 7400 Bay Road, University Center, Michigan 48710

The use of tourniquets for the control of hemorrhage from traumatic injury has been long debated. Opinions on the utility and safety of their use in this setting have alternated between strong endorsement and outright vilification of the device, with each of the camps backing up their contentions with varying levels of anecdotal evidence. The debate is largely fueled by experiences of military surgeons during wartime and the results have changed with changing times, differing systems and circumstances in which they have been utilized. Review of the evidence available in the English language medical literature seems to indicate that while neither camp is entirely correct, neither seems to be entirely without merit. The preservation of life- even at the potential expense of a limb- should without a doubt take precedence, but this should not lead to the abandonment of all possible efforts to minimize the length of time that the tourniquet is in place and the thereby reduce the attendant risk of complications.

The literature regarding tourniquets, their use, outcomes and complications was collected by a literature search of various pertinent databases. These included PubMed/MEDLINE, Ovid, EBSCOHost, and CINAHL utilizing keywords including, but not limited to, "tourniquet", "extremity", "hemorrhage", "bleeding", "combat", etc. The retrieved articles were assessed for pertinent information and the references they cited were accessed and reviewed to minimize the chance of pertinent sources being overlooked.

Extremity hemorrhage remains a common and significant cause of preventable trauma fatalities, both in the civilian world and the military theater, accounting for approximately 9% of fatalities in military actions. [1-7] Dorlac et al reported on preventable fatalities involving isolated extremity wounds presenting to two civilian trauma centers, and found that they occurred as 0.02% (N = 14) of the traumas seen at the facilities, with 50% caused by gunshot wounds and the remainder due to lacerations or stab wounds. Eight of the patients in this group had injuries that would have potentially benefited from management with a tourniquet.[8] Rocko et al reported on similar injuries, discussing eight cases where earlier attempts as hemorrhage control might have resulted in patient survival.[9]

The frequency of significant vascular injury from penetrating trauma among military personnel has been reported by Rasmussen *et al *as 6.6% (N = 209). These were casualties from Operation Iraqi Freedom treated at the Air Force Theater Hospital at Balad Air Base, Iraq with 79% (N = 166) of those cases involving the vasculature of the extremities, with the majority of these patient reaching definitive care in under an hour. [10] This is in stark contrast to many of the previous experiences with tourniquets, which indicates why an understanding of these circumstances is important in comprehending why the opinions that are held about tourniquets exist and what they mean for the current practitioner.

The first use of a tourniquet to attenuate hemorrhage from injury is not known with absolute certainty but the existence of similar devices has been described back to at least the Greeks. [11] Galen, the best known of the Roman surgeons, criticized the use of tourniquets as simply forcing more blood from a wound and this opinion was still repeated many years, even centuries, later by other authors. [12] This is likely based upon observation of patients with tourniquets that are applied with insufficient pressure to compress the artery while restricting the venous drainage of the affected limb.

The famous medieval physician de Chauliac described constricting bands for the reduction of pain and control of hemorrhage during amputation in 1586 and Ambrose Pare was noted to employ a similar technique. [13] The use of a triple band tourniquet system during amputations was attributed to Leonardo Botallo in the 16^th ^century, and the use of tourniquets under similar circumstances was described by von Gersdoff in his *Feldtbuch der Wundtartzney *("Field Manual of Wound Medicine") published in 1517. Wilhelm Fabry first described the basis for what most envision today as a "tourniquet", namely a device employing a windlass in 1593. [14]

A French surgeon at the Siege of Besancon in 1674 by the name of Etienne Morel was described as employing a rudimentary tourniquet during combat medical care. [15,16] A "screw compressor" was pictured in Johannes Scultetus' surgery text during the 17^th ^century, but this design was apparently limited in its utility due to issues with slippage and other factors. [17] The problem with slipping was reduced by Petit with his improved design that was introduced in the early 18^th ^century and allowed it to utilized further up on the limb. [18] Petit is also the source of the term "tourniquet" which he derived from the French verb for "to turn" (tourner). [19]

Much of the early criticism of the use of tourniquets stemmed from the delayed access to definitive care on the battlefield in many conflicts. MacLeod's treatise on the Crimean War questioned the benefit of tourniquets due to the seemingly insignificant number of vascular injuries that were seen in that conflict. This is more likely the result of those who would have benefited exsanguinating on the field while the battle was still ongoing and therefore never being seen by a surgeon, as he himself more or less stated. [20] This is a major issue with many of the early writings that contributed to attitudes towards tourniquet use in that lack of effective evacuation of wounded soldiers proved to make the statistics provided and outcomes cited, at best, of dubious value and, at worse, useless as a reference for decision making. In effect, the tourniquet bore more than its fair share of the blame for negative outcomes stemming from multiple factors including poor planning, lack of education of troops about the proper care of wounds, and the marginal medical logistics that all conspired to yield less than optimal results.

The American Civil War provided even more evidence of the dire consequences of failing to prepare for massive numbers of wounded soldiers. Surgeons were often seriously lacking in any experience dealing with traumatic injuries, let alone that of a recent nature sufficient to maintain skills. The variability of entry level training of physicians was also so great as to make broad characterizations of it is nearly impossible, [19] and the lessons of prior combat surgeons- as questionable as some of them may be from our current perspective- on the European continent seldom was known to the average military surgeon during the Civil War. The appreciable lack of medics was also a contributing factor, despite Letterman's establishment of an Union military ambulance system on the Napoleonic model, leaving men with minimal, if any, first aid training laying for hours, or even days in a few cases, on a battlefield. Gross addressed this in his 1861 text, where he implied that the supplies for a crude tourniquet should be part of the kit for every soldier, and the instructions on their use be provided, lest the soldiers "perish simply from their own ignorance". [19] Both in the *Manual of Field Surgery *and his later work *A System of Surgery*, Gross was highly critical of his fellow surgeons and laid the blame for the demise of many soldiers squarely at their feet: "I do not envy the man his feelings who. through ignorance, inattention, or indecision, allows his patient to perish from loss of blood when he ought to have saved him." [21]

The use of tourniquets, both improvised and those of professional design (most notably that of Petit) under circumstances where surgical intervention- and admittedly a crude form by modern standards- could not be counted upon for hours or longer proved to be less than desirable from the standpoint of limb salvage. Even in the face of severe pain associated with prolonged limb ischemia, many of the soldiers were loathe to loosen or remove a tourniquet for fear of further bleeding: "Very many of these wounded came into the hospital with extemporaneous tourniquets tightly applied, and their hands and forearms swollen and livid in consequence. This dread of hemorrhage is simply another proof of the inexperience of the troops." [22] Similar fear of recurrent bleeding is still common among troops today although the issue could likely be lessened through better education of soldiers about the nature of war wounds.

The excessive and inappropriate use of tourniquets by insufficiently trained and frightened soldiers on the battlefields of the Civil War led many surgeons to decry their use altogether. This included such extreme stances as that it was "far safer to leave the wound to nature, without any attempt to arrest the flow of blood than depend upon the common army tourniquet" as was attributed to one surgeon who was present at the Battle of Bull Run (Manassas). [23] This attitude of course is the result of the frequent amputations that followed such battles and the use of tourniquets. However it is also the opinion of someone who fails to taken into account the role the system in which tourniquets were being utilized played in the development of gangrene and ischemic complications. Given that after the first battle, some wounded men were left on the battlefield for days before evacuation few modern parallels can be drawn. The outcome of both battles, a poorly structured ambulance corps, and other factors that provoked a disastrous outcome for the casualties led to the reform of the medical operations of both sides. The improvements were demonstrated at the Battle of Antietam later that same year which is considered by most historians to be the turning point of the Civil War in regards to medical care. [24]

While the overwhelming opinion of surgeons towards the use of tourniquets was negative, little evidence beyond anecdotal opinions exists on which to judge the rate of tourniquet induced complications resulting in amputation that would have not have otherwise occurred.[21] The few sources that do cite data rely upon the questionable statistics that were included in MacLeod's Crimean War history, thereby grossly underestimating the frequency of vascular injury. Confederate Surgeon General Chisolm admitted in his text, while attempting to discourage the use of tourniquets that when vascular injuries do occur, the patient often exsanguinates so quickly that intervention is "of little avail".[25] Thus, he blatantly disregarded the most obvious- and probably least debatable- indication for the use of tourniquets, that being the attempted preservation of life at any cost, including the sacrifice of an extremity.

The "disasters" that stemmed from such hindrances even provoked knee-jerk reactions that may well have cost soldiers their lives for little benefit, such as that proffered by Tuffier who was a respected surgeon with the French Army during the First World War. He recommended that as soon as ambulance crews encountered a patient with a tourniquet in place that it be removed. [26] Given that the patient most likely had been laying in "no man's land" for many hours with the tourniquet in place, the likelihood of the immediate removal of the tourniquet offering any improvement in the outcome for the limb is highly suspect and the possibility of provoking further hemorrhage would more likely be the result.

One of the most dramatic, and retrospectively shortsighted, denunciations of tourniquets can be found in *Injuries and Diseases of War*, which was a British manual that was reprinted in the United States in 1918:

"The systematic use of the elastic tourniquet cannot be too severely condemned. The employment of it, except as a temporary measure during an operation, usually indicates that the person using it is quite ignorant both of how to stop bleeding properly and also of the danger to life and limb caused by the tourniquet . . . If an orderly has applied a tourniquet, it is the duty of the medical officer who first sees the patient to remove it at once, and to examine the limb so as to ascertain whether there is any bleeding at all, and if there is, to use proper measures for its arrest."

Once again, the admonition never to allow a tourniquet to be left in place beyond the prehospital phase of care was repeated due to the risk of pain, infection and amputation.[27] While immediate conversion to less aggressive measures of hemorrhage control are optimal, such across the board advice is most likely the source of the modern day hesitancy to utilize tourniquets in any manner. One must question whether this belief arose as the product of a seriously flawed system of medical care, as obviously existed, rather than an inherent flaw in the idea behind the use of tourniquets.

More useful information regarding tourniquets, still largely applicable, was provided by Tuttle[28]:

1. Never cover over or bandage a tourniquet.

2. Write plainly on the emergency medical tag the word 'tourniquet.'

3. If the injured man is conscious, he should be instructed to tell every medical officer with whom he comes in contact that he has a tourniquet on.

Tuttle also emphasized the use of arterial "pressure points" to "buy time" in which other methods of control can be employed, including the application of a tourniquet.

Bailey in his seminal text on war surgery, published during the Second World War, gave a great deal of attention to the subject of tourniquets and indicated that tourniquets have a place in management of arterial bleeding that fails to respond to other interventions. He also suggested the preemptive application of a loosely applied tourniquet in cases of secondary hemorrhage and their use to provide a bloodless surgical field.[29] The latter use has become commonplace in hospitals around the world today, through the application of pneumatic tourniquets in orthopedic procedures.

The text also reinforced the need for proper and early identification of those patients with tourniquets in place, through proper labeling. Increased bleeding from insufficient pressure, as mentioned above, was also pointed out as a potential hazard of the use of tourniquets, while at the same time the use of excessive pressure was discouraged due to the risk of local skin damage and other complications. A quote from Bailey is one of the best summations of the subject matter found anywhere, stating that a tourniquet should be "regarded with respect because of the damage it may cause, and with reverence because of the lives it undoubtedly saves. It is not to be used lightly in every case of a bleeding wound, but applied courageously when life is in danger." [29]

During the preparations for the invasion of Normandy in 1944, the Allied Forces medical personnel were provided with a text that included instructions for the care of vascular injuries. Part of this advice was a statement that any limb requiring a tourniquet that remained in place during evacuation would most likely require amputation but that any suspected or known injury to the blood vessels was sufficient reason to send a tourniquet along with the patient during transport should the need for it arise. [30]

One of the best articles with the sole purpose of examining issues related to tourniquet use in a large group was written during WWII by Wolff and Adkins which looked at a series of over 200 wounded servicemen who had tourniquets applied. The authors were critical of the strap and buckle tourniquet issued by the Army, due to its inadequate occlusive pressures and the tendency to dig into tissues. They also described occlusive times of up to six hours with no clinically significant damage depending on which extremity was involved and the environmental conditions; anecdotal reports from cases occurring during the wintertime indicated that cold temperatures and resultant cooling of the affected limb might lead to minimal negative effect on the limb despite prolonged ischemic times. Wolff and Adkins rank among the staunchest advocates of the use of tourniquets in combat casualty care during WWII. They firmly denounced the fears of damage stemming solely from the use of the tourniquet, finding not a single case of gangrene directly attributable to the use of such a device alone, nor were thromboembolic events, skin damage, excessive edema or nerve damage reported during the postoperative management of any of their patients.[31]

The United States Army Medical Department in a review of the medical services of World War II stated that soldiers frequently misused tourniquets, failed to alert staff at aid stations of their presence and otherwise contributed to negative outcomes stemming from the use of tourniquets. This was such a widespread problem that their use was restricted in one unit that the senior surgeon ordered that the only reason for the use of such a device was for the control of "active spurting hemorrhage from a major artery". The directive was also issued to reinforce the proper documentation of the placement of a tourniquet to allow rapid notification of upper echelon personnel. [3,32] The early advice to loosen the tourniquet every 30 minutes to allow perfusion of the limb via collateral circulation due to the fact that the practice put a patient at risk of bleeding to death by slow degrees was also replaced with orders that a tourniquet that should only be removed by a medical officer. This opinion continues to be common practice today.[26,33]

It should be noted, for the sake of full disclosure, that perhaps not all of the blame for poor outcomes should be trained at the tourniquet or the men applying them, or the system in which they functioned- although admittedly the delays in access to operative intervention undoubtedly played a role as did the inappropriate battlefield care of the wounded. The operative techniques employed by military surgeons for vascular trauma suffered, secondary to both the case volume and a failure of the military medical system to learn the lessons of prior conflicts. Ligation of arteries was a common practice especially during the early stages of the war, and one that produced a high rate of gangrene as documented in the literature.[34,35] This is in no way a condemnation or an attack on the skill and dedication of the surgeons who served the militaries of all the combatant nations, but rather another sad example of history repeating itself when appropriate lessons are either not learned or not applied. This is supported by the fact that as the war progressed, amputation rates decreased as surgeons gained experience with the injuries common on the battlefield, in which they were not well educated prior to their deployment due to oversight on the part of their commanders.

One of the most notable military surgeons deployed to Korea was Dr. Carl Hughes and his publications on combat related vascular trauma provide valuable insight into the progress that was made during the intervening years between then end of WWII and the start of hostilities in Korea. While he was openly critical of the manner in which many tourniquets during that conflict were applied,[36] he has been quoted as recently saying "I do not recall ever seeing limb loss as a result of a tourniquet. They were important, even life saving, in Korea. Successful use of the tourniquet depends on what it is made of, and how it is applied". [24] The recounted experiences of Jahnke, Hughes and others during this time also serve to dispel the myth that a tourniquet is invariably associated with amputations, while reinforcing the role that evacuation delays played in amputation following tourniquet application, as more attempts were made at limb salvage through vascular repair techniques. [37,38]

Improvised tourniquets were commonplace during the conflict in Vietnam and their use by medics was deemed to be more judicious by some of the attending surgeons with at least one (JE Hutton) attributing this to the fact that "most of our medics were college graduates, were bright and well trained." [24] Also the preemptive use of fasciotomies became more common as a step in combating compartment syndrome associated with prolonged tourniquet use, which was much less frequent than encountered in any previous war due to the unprecedented use of helicopters as a primary means of casualty evacuation. It has been said repeatedly before that many soldiers wounded in southeast Asia owe their lives to the "Dustoff" crews (that is, United States Army medical evacuation helicopter crews), but perhaps this is better rephrased as many of the wounded owe their lives *and their limbs *to these brave souls.

However, not all surgical authorities serving in the Vietnam War have such uniformly positive assessments of the use of tourniquets. Dr. Norman Rich reported the anecdotal case of an upper arm injury that was bleeding because of the presence of the tourniquet, the removal of which staunched the hemorrhage.[24] He later went on to state that the necessity of the use of tourniquets in Vietnam was an infrequent occurrence.[39] Regardless of their stance on this issue, the dedication, resourcefulness and talents of the Vietnam medical personnel are largely responsible for the current era of limb salvage that stems from rapid evacuation and early and aggressive operative intervention.[10]

Until recent years, the staunchest supporters of the use of tourniquets were the Israeli Defense Forces (IDF), and widespread use by the IDF yielded some of the best data available on the complications associated with modern battlefield use of tourniquets. Despite what may best be described as overzealous utilization by soldiers, there has been a paucity of complications reported and those that have occurred are most often temporary in nature. The isolated incidents of permanent complications were associated with prolonged use of a tourniquet and serve as further evidence that the opinion of tourniquets as invariably damaging to the limb is misguided. [40]

Despite the methodological misgivings of a few [41], the Lakstein study- particularly when considered along with other reports that are discussed elsewhere in this paper- shows that tourniquets are an acceptably safe and effective means of hemorrhage control on the modern battlefield where rapid access to definitive intervention is the rule, rather than the exception. The use of tourniquets amongst special operations troops has been particularly widespread in the US military for quite some time, and the experiences of the Rangers in Somalia provide additional evidence of the benefits offered by the use of tourniquets by military personnel.[5] Other special operations units also encourage tourniquet use for hemorrhage control in combat situations. [42-44]

The aggressive use of tourniquets among trauma patients transported to the Air Force Theater Hospital at Balad led to no cases of serious complication, even when taking into account infrequent cases of inappropriate use (in the setting of no major arterial injury). This is presumably due to the rapid evacuation of casualties and the short time to operative intervention, often less than one hour [10]. Chambers reported even more rapid arrival of patients at facilities with surgical capability in his paper reporting the experiences of the United States Marine Corps' Forward Resuscitative Surgical System. [45] This contrasts with the average time for similar cases in the Vietnam War where the time to operation for a majority of patients was variously reported as 90 minutes for all patients with ballistic injury, [46] up to five and a half hours for injuries to the popliteal artery treated aboard a United States Navy hospital ship [47]. Regardless of which study is relied upon, there was an improvement over the average of 9.2 hours reported in the Korean War [48].

The data from Balad is comparable to the earlier report based upon patients treated at Walter Reed Army Medical Center, [49] but rate of vascular injury is significantly higher than reports that looked at rates of similar injury among military personnel in Vietnam who survived to be treated at military medical facilities, which routinely reported rates of between 2–3%. [50,51] In the report from Iraq, Rasmussen and his colleagues suggested several possible reasons for the disparity, including better documentation of vascular trauma among casualties and increased survival of patients with peripheral vascular trauma due to improvements in body armor lessening mortality from thoracoabdominal trauma. [10]

Walters and Mabry stated that the proper use of tourniquets could potentially prevent seven of every 100 deaths due to combat related injury. [33,52] A similar positive attitude can be found in many of the recent articles dealing with tourniquets. The review by Welling et al contains several anecdotal statements from experienced military physicians who indicate the utility of the tourniquets in modern combat and the lack of significant complications.[17] The military's *Emergency War Surgery *text explicitly supports the use of tourniquets in combat, encourages risk to benefits assessment in any setting other than active combat but admonishing that no life under should be lost due to hesitance from perceived risks of limb loss.[53] The author of this paper has personal anecdotal experience with the successful prehospital use of a blood pressure cuff to control arterial bleeding while pressure dressings were applied to a combative patient with an amputation of the hand secondary to a lawnmower accident.

The fact that many of those who perish in combat do so rapidly and before evacuation to combat hospitals or aid stations can be accomplished, with the majority being due to hemorrhage with the source of the bleeding in many cases being an extremity wound. As Welling pointed out, the only chance to save these lives rests with the medics and the soldiers themselves [17]. Given the nature of care under fire- the risks to the caregiver, the need for rapid extrication of the wounded to cover, and the frequency of mass casualty events- to express expectation that direct pressure can be utilized as a first line response under such circumstances is to speak to one's lack of awareness of the circumstances faced by the medic and the wounded alike. It is for this reason that the United States military has emphasized the use of tourniquets during the prehospital care of wounded and sought out a design that was able to be self-applied by a wounded soldier. [43,54] Not only does a properly applied tourniquet control hemorrhage [55-57] and allow time for the gravely wounded to reach definitive care, they also provide the chance for the medic to render care to other injured persons. Such practices may also facilitate transport of casualties, especially in the case of multiple victims.

The control of hemorrhage in the civilian setting is less fraught with serious risk to the first responder and therefore is much more able to follow the traditional stepwise approach recommended by most authorities. The advice of Rich and Spencer, which includes packing of the wound with associated arterial hemorrhage, direct pressure and pressure dressings [39] is probably the best approach when sufficient manpower and safe circumstances to allow intervention by trained and skilled providers. Outside of situations necessitating expedient evacuation of casualties, the use of a tourniquet will be necessary only infrequently but should be considered in any case where hemorrhage is ongoing and life threatening. This approach is similar to that recommended by Aucar and Hirshberg, [58] as well as that recommended by the Advanced Trauma Life Support manuals [59,60], as well as the *US Army Survival Manual *[61] which is widely distributed to the general public through a civilian publisher.

However, the safest approach in the case of the marginally trained and inexperienced person with basic first aid training is probably to rely upon simple direct pressure or basic forms of pressure dressing. This is due to a lack of evidence that such persons can effectively recognize the need for a tourniquet and properly apply such a device- especially given the likely need to improvise under such circumstances. [9,62] This last point is illustrated by a case of femoral artery transection by broadhead arrow as the result of a deer hunting accident to which the author responded as an emergency medical technician. The victim's nephew had attempted to place a tourniquet made from the victim's belt prior to going for help. The patient was deceased due to blood loss at the time of the arrival of the author and his coworkers. It was determined that the bystander had improperly placed tourniquet distal to the injury and with insufficient force to be of any utility even if it were in a proper position.

Probably the strongest argument towards the broader use of tourniquets in the field is the experience of the United States military, [63-65] such as in Iraq where the combination of aggressive hemorrhage control and rapid transport has produced minimal complications associated to tourniquet use. [66-69] A few anecdotal reports of deaths that may have been preventable by the timely application of tourniquets for control of bleeding have also emerged from the battlefields of the Middle East and serve to point out that while improvements in care have been made, there are still cases that can be learned from.[70] While the tourniquets can not be given sole credit, their ability to allow those who would have otherwise bled out to receive the full benefit of modern trauma care as was described by the Balad vascular team and other- early thrombectomy and heparin administration along with vascular reconstruction or shunting and fasciotomy when necessary- can not be denied.

The use of tourniquet as a "stopgap" measure in combat [56]- with reassessment of the necessity of the tourniquet as soon as situational conditions allow- is part of the Tactical Combat Casualty Care course the United States Army conducts. [71,72] This emphasis on conversion to less aggressive means of hemorrhage control whenever possible may be one reason that reports from the Iraq theater of operations describe the presence of unnecessary tourniquets upon arrival at medical facilities as infrequent. This attitude has been incorporated into the military version of the Prehospital Trauma Life Support (PHTLS) manual, [73] which is widely used in the education not only of military personnel, but also in the education of tactical medics in the law enforcement community as well. Even some staunch opponents of the widespread use of tourniquets admit that the temporary use of tourniquets under tactical conditions or similar circumstances is acceptable to effect the safe extraction of the wounded party. [40,41]

The rapid employment of tourniquets may also provide an opportunity to improve the prognosis for those who might otherwise not receive care due to the severity of their injuries in a mass casualty situation where triage principles are applied. The expedient control of extremity hemorrhage may allow a few of these patients to survive long enough for them to be evacuated even when a medic may be forced to move on to another patient due to prioritization.[74] This is similar to techniques employed in damage control surgery- in both combat and civilian settings- where pneumatic tourniquets have been used in place of vascular clamps to allow the control of more immediately life threatening thoracic and abdominal injuries, as well as in isolated orthopedic cases prior to reconstruction or shunting. [66,67,75,76] It has also been utilized for the control of hemorrhage during ongoing emergency department resuscitation of combat casualties.[77] While the possibility of such a technique being utilized outside of a medical facility is speculative at this point, it might be worthy of further investigation to determine the feasibility and utility of such a recommendation.

The use of tourniquets, while beneficial to many of those wound in combat or with otherwise uncontrollable bleeding, is not without its hazards and potential complications. Any use of a tourniquet must be with full awareness of the risks involved and to brush these aside would be to abandon one of the basic tenets of evidence based medical practice.

Most of the complications stemming from tourniquet use are either the result of direct pressure on underlying tissues or the byproducts of ischemia distal to the site of application. While most of the complications that have been reported in association with their use (both for control of hemorrhage and as an adjunct to surgery) have been localized, there are systemic complications that can result including thromboembolic events [78], most notably pulmonary embolism; renal failure due to rhabdomyolysis [79-84]; lactic and respiratory acidosis, hyperkalemia, arrhythmias, and shock[85].

The use of tourniquets during elective surgery has led to reports of cardiac arrest secondary to circulatory overload in patients with poor cardiac reserve resulting from a functional increase in the circulating blood volume. This is likely to not be a factor in a hypovolemic trauma patient but may play a role in the case of a patient with underlying heart disease who is being fluid resuscitated with a tourniquet in place. Tourniquet removal postoperatively has produced transient increases in end-tidal carbon dioxide levels, and transient decreases in central venous pressure and blood pressure. The former may be of significance in a patient with head trauma, but the effect can be minimized through hyperventilation of the patient. Release of a tourniquet has also been described to induce brief systemic thrombolysis as a result of the stimulation of various anticoagulation mechanisms by ischemia.[86]

Localized complications have included pain, erythema or localized bullous skin lesions, nerve damage [78,87] from paresthesias to paralysis of the affected limb, vascular spasm, fracture of atheromatous plaque, muscle injury [88], gangrene and other infectious complications, edema, to compartment syndrome [78]. The nerve and muscle injuries may be transient or permanent in nature[89], although the latter is exceedingly uncommon in most settings today where tourniquets are utilized for hemorrhage control. This is due to a strong positive correlation between the length of time the tourniquet is in place and the rate and severity of complications that result.[39,90] A similar correlation exists with the amount of pressure produced by the tourniquet,[91,92] but this is mainly an issue with improvised tourniquets and those with a width of one inch or less. It should also be noted that patients with preexisting neuropathies, such as those associated with diabetes or alcohol abuse, appear to be at an increased risk of nerve injury,[93] and other factors may also serve to predispose patients to nerve related complications.

Complications of questionable association, due to a lack of corroborating clinical evidence in injured human subjects to support such claims, include the possible affects of inflammatory mediators on the gut mucosa following ischemia of a limb. This assertion was made by persons with a stated distrust of the use of tourniquets and was accompanied by an unsubstantiated claim that the use of a tourniquet in the hypotensive patient places the patient at a "considerable risk" of loss of life.[41] Such contentions are largely refuted by the volume of cases that have been recently entered into the literature as a result of current military operations without any indication that serious complications of a systemic or localized nature have been frequently associated with the short term (< 2 hrs) use of tourniquets for hemorrhage control. It is for this reason that until evidence supporting such claims of negative systemic outcomes stemming directly and without question from the use of tourniquets by properly trained and equipped medical professionals, the assertions to that effect must be viewed with a certain degree of skepticism.

Failure of a tourniquet is usually the result of insufficient pressure, but this can easily be prevented by reinforcing during the training of those who will be employing such devices that total arterial occlusion is the goal. There have been isolated cases reported among surgical patients where extreme calcification of the arteries prevented effective use of tourniquets for the establishment of a bloodless field.[94-96] This is unlikely however to be a significant factor in the use of tourniquets for hemorrhage control.

There are still several unanswered or only partially answered question regarding the use of tourniquets and the attendant complications, infrequent as they may be in current practice. These include the role of hypothermia [31,97-101] and agents such as antioxidants in minimizing muscle and nerve damage from ischemia. The former has already been demonstrated to be of benefit on a limited basis, with even a marginal (2–3 degrees Celsius) decrease in muscle temperature has been shown to be beneficial.[32] Further research into these aspects of trauma care, and others, are still needed and therefore should be encouraged.

The use of the tourniquet in hemorrhage control is likely to remain controversial for the near future, however given the best evidence available mandates serious reconsideration of the attitudes that we as a profession hold toward this practice. While there are potential risks involved in the utilization of tourniquets should not be overlooked, expeditious and clinically and/or situation appropriate application in the presence of potentially life threatening hemorrhage is in keeping not only with the standards of the medical professions, but accordingly so with the best interests of the patient.

Based upon the best evidence available from the literature, the following conclusions are drawn:

- Emergency medical personnel, both civilian and military, should be trained in and equipped for the proper use of tourniquets; the focus of first aid training for civilian populations should continue to deemphasize their use and focus instead on early medical assistance and the use of direct pressure to control hemorrhage.

- No patient should exsanguinate from an extremity wound because of the hesitance of a medical professional to utilize a tourniquet to control bleeding due to fear of potential complications.

- In circumstances- such as combat (or the civilian equivalent thereof), high risk of building collapse, fire, or explosions- where expedient movement of the patient is necessary for the safety of the patient and the caregivers, the use of a tourniquet is appropriate to gain control of life threatening hemorrhage

- The existence of a mass casualty incident may be an indication for the use of tourniquets for temporary control of hemorrhage while the situation is brought under control.

- The need for a tourniquet applied to allow movement of a wounded person or during a mass casualty incident should be reevaluated at the earliest possible time;

- The mere presence of an amputation with hemorrhage does not necessitate the use of a tourniquet; most bleeding from such injuries are controllable through use of direct pressure, elevation and packing of the wound. If these actions do not achieve hemostasis, then the use of a tourniquet is indicated.

- Tourniquets may be placed proximal to the site of uncontrollable bleeding around an impaled object; under no circumstances should the tourniquet be applied over the impaled object.

- Tourniquets should not be applied over joints, or over clothing. It should also be at least 3–5 centimeters from the wound margins. The rule of the thumb the author used when teaching was to place it the width of the palm of a hand proximal to the wound whenever possible, as this provides an easy frame of reference.

- Any limb with an applied tourniquet should be fully exposed with removal of all clothing, and the tourniquet should never be covered with an form of bandage. The patient should be clearly marked so as the presence of a tourniquet will be know, along with the time it was placed. It may also be advisable to instruct a conscious patient to tell every medical provider they come in contact with about the presence of a tourniquet.

- Continued bleeding (other than medullary oozing from fracture bones) distal to the site of the tourniquet is a sign of insufficient pressure and a need to tighten the tourniquet further.

- A tourniquet should not be loosened in any patient with obvious signs of shock, amputation that necessitated use of such a device to control bleeding, recurrent hemorrhage upon release of the tourniquet or any case where the hemorrhage associated with the wound would be expected to be uncontrollable by any other means.

- Any tourniquet that has been in place for more than six hours should be left in place until arrival at a facility capable of definitive care.
